# Three-Component Subunit Vaccine Induces Protective Immunity Against *Mycoplasma pneumoniae* in Mice

**DOI:** 10.3390/vaccines14040330

**Published:** 2026-04-07

**Authors:** Han Wang, Tiantian Wang, Zhuoran Hou, Ruixi Liu, Qianhui Liu, Zhu Zhou, Bin Zhang, Xuchen Hou, Lu Li, Jun Wu, Bo Liu

**Affiliations:** 1 National Key Laboratory of Advanced Biotechnology, Academy of Military Medical Sciences, Beijing 100071, China; wanghan010811@163.com (H.W.); con_an@126.com (T.W.); houzhuoranzzz@126.com (Z.H.); liuruixi0521@outlook.com (R.L.); 2024608032019@stu.zafu.edu.cn (Q.L.); zhuzhou0912@163.com (Z.Z.); zhangbin@bmi.ac.cn (B.Z.); hoxuch@163.com (X.H.); ll13051378779@163.com (L.L.); 2 Tianjin Key Laboratory of Agricultural Animal Breeding and Healthy Husbandry, College of Animal Science and Veterinary Medicine, Tianjin Agricultural University, Tianjin 300392, China; 3 College of Animal Science and Technology, Zhejiang Agriculture and Forestry University, Hangzhou 311300, China; 4 School of Life Sciences and Medical Enginering, Anhui University, Hefei 230601, China

**Keywords:** *Mycoplasma pneumoniae*, recombinant subunit vaccine, multi-component, P1, P40/90, CARDS toxin

## Abstract

Background: *Mycoplasma pneumoniae* (MP) is a major cause of respiratory tract infections in children and adolescents. Currently, there is no licensed vaccine, underscoring the urgent need for the development of safe and effective vaccines. Objective: The aim of this study is to develop a recombinant subunit vaccine candidate incorporating three antigens: the P1 protein, the P40/90 complex, and a detoxified mutant of community-acquired respiratory distress syndrome toxin. The protective efficacy of this vaccine candidate was also evaluated. Methods: Target genes were codon-optimized for expression in *E. coli*, and the recombinant proteins were successfully expressed and purified. The low-toxicity CARDS toxin mutant was screened based on TNF-α secretion levels in stimulated RAW264.7 cells. A three-component vaccine composed of P1, P40/90, and the mutant CARDS toxin was formulated and adjuvanted with either Al(OH)_3_ alone or in combination with CpG. Mice were immunized, and immunogenicity was assessed by measuring antigen-specific IgG antibody titers. Protective efficacy was evaluated following challenge by analyzing lung histopathology, bacterial load, and inflammatory cytokine levels. Results: Seven high-purity recombinant proteins were successfully produced, including P1, the P40/90 complex, wild-type CARDS toxin, and four CARDS toxin mutants (E132A, E132Q, H36A, R10A). The E132A mutant was selected due to its significantly reduced toxicity while retaining immunogenicity. The three-component vaccine effectively elicited antibody responses against each of the included antigens. After three immunizations, IgG antibody titers in all groups reached approximately 10^4^. Immunized mice showed markedly reduced pulmonary pathology scores (control group: 2 or 2.67; immunized groups: 1.67, 1.33, and 0) and significantly decreased bacterial loads in lung tissue (control: 30.11 ± 10.40 cp/μL; immunized groups: 20.72 ± 4.37 cp/μL and 8.51 ± 8.32 cp/μL). Furthermore, the group receiving the alum + CpG adjuvant exhibited approximately a 10-fold higher antibody response compared with the alum-only group, indicating enhanced protective efficacy. Conclusions: The three-component candidate vaccine, MPtriV, adjuvanted with Al(OH)_3_ + CpG, demonstrates promising immunogenicity, safety, and protective efficacy against *Mycoplasma pneumoniae* infection, providing a viable strategy and experimental foundation for the development of MP subunit vaccines.

## 1. Introduction

*Mycoplasma pneumoniae* (MP) is a minimal, cell wall-less prokaryote capable of independent life and is a major causative agent of acute respiratory infections worldwide [1]. It is primarily transmitted via respiratory droplets, demonstrating high infectivity and a tendency for outbreaks among children, adolescents, and individuals in crowded settings [1]. MP infections have a long incubation period and typically present with symptoms such as cough, sore throat, and fever. In some cases, the infection may progress to bronchitis or pneumonia, and in severe instances, lead to life-threatening extrapulmonary complications affecting the cardiovascular or nervous systems, or even cause autoimmune diseases [2,3]. Furthermore, MP can establish long-term colonization in vivo, leading to chronic or recurrent infections, which pose significant challenges for clinical prevention and control [4]. Current treatment primarily involves macrolide or quinolone antibiotics; however, the widespread emergence of resistant strains is reducing the efficacy of these treatments, and antibiotics cannot prevent transmission from the source [4]. Therefore, the development of a safe and effective MP vaccine is essential for controlling infections and holds substantial public health importance.

The pathogenicity of MP results from multiple synergistic factors, which can be summarized into three main mechanisms: First, adhesion damage, where surface adhesins specifically bind to host respiratory epithelial cells, disrupting epithelial integrity and compromising the mucosal barrier function [5]. Second, toxin-mediated damage, where the secreted community-acquired respiratory distress syndrome (CARDS) toxin induces host cell inflammatory responses, promoting the release of cytokines and causing tissue damage [6]. Third, immune-mediated damage, where MP infection triggers aberrant host immune responses, leading to the release of excessive inflammatory mediators that further exacerbate respiratory and lung tissue injury [7]. Adhesion is a prerequisite for MP colonization and pathogenesis, while CARDS toxin is the central virulence factor responsible for inducing severe inflammatory reactions and tissue damage.

This study selected the P1 protein, the P40/90 complex, and CARDS toxin as vaccine antigens due to their central roles in *Mycoplasma pneumoniae* (MP) pathogenesis and immunity [8,9]. The P1 protein, a major adhesin on the MP surface (approximately 170 kDa), specifically binds to neuraminic acid receptors on host respiratory epithelial cells, making it essential for colonization and pathogenesis. P1 is highly antigenic and induces specific neutralizing antibodies that inhibit MP adhesion, making it one of the most widely used antigen targets in MP vaccine development. The P40/90 complex, consisting of the P40 and P90 proteins, assists P1 in enhancing adherence to host cells and also exhibits strong antigenicity, inducing immune responses that complement P1 in providing immune protection. CARDS toxin, the sole virulence toxin of MP, is not only highly antigenic, inducing antibodies that neutralize its toxic effects [10], but its “immune toxicity”—characterized by excessive induction of inflammatory cytokines—likely contributes to the severe lung injury seen in MP infection [11,12]. Consequently, incorporating CARDS toxin into a vaccine involves reducing its immune toxicity through site-directed mutagenesis, while preserving or even enhancing its immunogenicity. This approach addresses safety concerns associated with using wild-type CARDS toxin and, through its antigenicity, targets the toxin-mediated damage pathway. Combined with the adhesion-blocking effects of P1 and P40/90, this strategy can significantly enhance the vaccine’s immune protection. The synergistic action of these three components is expected to address key pathogenic steps, namely adhesion and toxin-mediated damage, ensuring robust immune protection.

Current research on *Mycoplasma pneumoniae* (MP) vaccines remains a hotspot, but no vaccine has yet been approved for use [9]. Research efforts primarily focus on three categories: First, inactivated and live-attenuated vaccines. Inactivated vaccines are simple to prepare but exhibit weak immunogenicity, requiring multiple doses and potentially causing adverse reactions, such as immune enhancement [13]. Live-attenuated vaccines elicit strong, broad immune responses but pose significant safety risks, limiting their clinical applicability. Second, subunit vaccines, which target key MP antigenic proteins. These vaccines offer advantages, including well-defined components, high safety, and strong specificity, making them the primary focus of current MP vaccine research. However, most existing studies concentrate on single or dual antigens, resulting in a narrow immune protection spectrum and limited efficacy [14,15,16,17]. Third, nucleic acid and live vector vaccines, which can induce comprehensive immune responses and allow rapid preparation, though they are still in the early stages of research, requiring further validation of their immunogenicity, safety, and clinical efficacy [18]. In light of these challenges, this study selected P1, the P40/90 complex, and CARDS toxin as core antigens, modified CARDS toxin via site-directed mutagenesis to reduce its toxicity, and formulated a three-component recombinant subunit vaccine with appropriate adjuvants. The goal was to enhance the vaccine’s safety, immunogenicity, and protective efficacy, thereby providing new insights and experimental evidence for MP vaccine development.

Based on the above considerations, we hypothesized that a three-component recombinant subunit vaccine incorporating P1, P40/90, and a detoxified CARDS toxin mutant would elicit a broader and more effective protective immune response against *Mycoplasma pneumoniae* compared to single-antigen formulations. In addition, we hypothesized that site-directed mutagenesis of CARDS toxin could significantly reduce its toxicity while preserving its immunogenicity, and that the combination of Al(OH)_3_ and CpG adjuvants would further enhance the overall immunogenicity and protective efficacy of the vaccine.

## 2. Materials and Methods

### 2.1. Strains and Materials

*Escherichia coli* Trans5α competent cells were obtained from Beijing TransGen Biotech Co., Ltd. (Beijing, China), and *E. coli* BL21 (DE3) competent cells were sourced from Beijing Tiangen Biotech Co., Ltd. (Beijing, China) Yeast extract, agar, and tryptone were purchased from OXOID (Basingstoke, UK). Kanamycin was acquired from Beijing Solarbio Science & Technology Co., Ltd. (Beijing, China) The Al(OH)_3_ adjuvant was obtained from Croda Denmark A/S, (Hørsholm, Denmark) and CpG was purchased from Beijing Liuyihe Company (Beijing, China).

### 2.2. Recombinant Protein Design, Expression, and Purification

**Figure 1:**
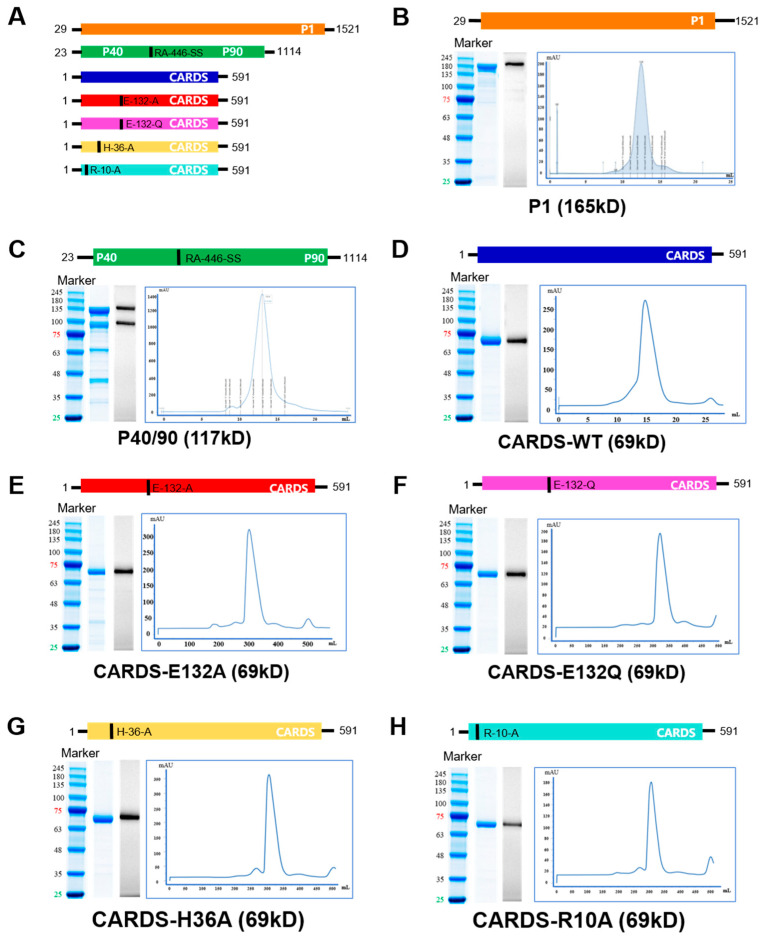
Recombinant protein design, construction, and purity validation. (**A**) Schematic diagram of recombinant protein constructs. Mutation sites are indicated by short black lines. (**B**–**H**) SDS-PAGE, Western blot, and Superdex™ 200 Increase 10/300 GL purification results for recombinant proteins. (**B**) P1 (~165 kDa); (**C**) P40/90 complex (~117 kDa); (**D**) CARDS-WT (~69 kDa); (**E**) CARDS-E132A (~69 kDa); (**F**) CARDS-E132Q (~69 kDa); (**G**) CARDS-H36A (~69 kDa); (**H**) CARDS-R10A (~69 kDa). Left lane: protein marker; right lane: purified recombinant protein.

The coding sequences for P1 (GenBank I D: X06871), P40 (GenBank ID: AF125204), P90 (GenBank ID: AF125205), and CARDS-WT (GenBank ID: AY189944) were retrieved from the NCBI database based on the *Mycoplasma pneumoniae* standard strain ATCC M129. The genes were codon-optimized for expression in *E. coli*. Additionally, four site-directed mutants targeting key toxic residues of the CARDS protein (E132A, E132Q, H36A, R10A) were designed [12], as depicted in Figure 1A. The mutant genes were generated using site-directed mutagenesis with specifically designed primers.

The optimized P1, P40/90, CARDS-WT, and four mutant genes were individually cloned into the pET-30a vector to construct recombinant expression plasmids. After sequencing to confirm correct gene sequences and insertion orientation, the plasmids were transformed into *E. coli* BL21 (DE3) competent cells. Single colonies were inoculated into Medium 1 (per liter: 20 g yeast extract, 10 g tryptone, 50 mL PB buffer, pH 7.0) supplemented with 50 μg/mL kanamycin. Cultures were grown at 37 °C with shaking until the optical density (OD) at 600 nm reached 0.6–0.8, induced with IPTG (final concentration 1 mmol/L) at 20 °C for 20–24 h, and then harvested by centrifugation.

Bacterial pellets were resuspended in deionized water at a 1:30 (mass/volume) ratio, sonicated, and then centrifuged. The supernatant was collected and purified using Ni Sepharose 6 Fast Flow (Cytiva, Uppsala, Sweden). Target proteins were eluted with elution buffer containing 150 mmol/L imidazole. The eluted protein fractions were further purified by size exclusion chromatography using a Superdex™ 200 Increase 10/300 GL column (Cytiva). Protein concentrations were determined using the BCA assay, and protein purity was assessed by SDS-PAGE (10% separating gel) followed by Coomassie Brilliant Blue staining.

### 2.3. Recombinant Protein Purity Analysis

The purity of recombinant proteins (P1, P40/90, CARDS-WT, and CARDS mutants) was analyzed by High-Performance Liquid Chromatography (HPLC) using a TSKgel G2000SWXL column (Tosoh Bioscience, Tokyo, Japan). A 50 μL sample of purified protein was loaded onto the column, which was pre-equilibrated with PBS buffer (pH 7.4) containing 0.5‰ Tween 80. Elution was carried out at a flow rate of 0.4 mL/min, and absorbance was monitored at 214 nm. Protein purity was determined by calculating the peak area percentage of the main peak.

### 2.4. Screening for Low-Toxicity Community-Acquired Respiratory Distress Syndrome (CARDS) Toxin Mutants

The toxicity of wild-type and mutant CARDS proteins was assessed using RAW264.7 cells (mouse macrophage-like cells). RAW264.7 cells were seeded in 96-well plates at a density of 5 × 10^4^ cells/well and cultured for 24 h [11]. Cells were then treated with seven concentrations (0, 5, 10, 25, 50, 75, 100 μg/mL) of CARDS-WT or each mutant protein in duplicate wells and incubated for 24 h at 37 °C in a 5% CO_2_ atmosphere. Cell culture supernatants were collected, and TNF-α levels were measured by ELISA (R&D Systems, Inc., Minneapolis, MN, USA) to identify mutants with significantly reduced toxicity, as indicated by decreased cytokine secretion.

### 2.5. Immunogenicity Assessment of Antigen Proteins

Female BALB/c mice (6–8 weeks old) were obtained from Beijing Vital River Laboratory Animal Technology Co., Ltd. (Beijing, China) and housed in the SPF facility at the Laboratory Animal Center, Academy of Military Medical Sciences. All animal experiments were approved by the Institutional Animal Care and Use Committee (Ethics No.: IACUC-2024-039). Mice had ad libitum access to food and water and were acclimatized for one week prior to the start of the experiments.

For the evaluation of single antigen immunogenicity, 25 mice were randomly divided into 5 groups (n = 5 per group): Saline control, P1 protein, P40/90 protein, CARDS-WT protein, and CARDS-Mut protein. Mice were immunized intramuscularly (i.m.) with 100 μL per dose, containing 5 μg of the respective antigen protein, adjuvanted with 100 μg Al(OH)_3_. Immunizations were administered on days 0, 14, and 28. Sera were collected 14 days after the final immunization (day 42) and stored at −20 °C.

To evaluate the immunogenicity of the three-component vaccines, 48 BALB/c mice were randomly divided into 6 groups (n = 8 per group). The experimental groups received vaccines containing P1 + P40/90 + CARDS-WT (MPtriVa) or P1 + P40/90 + CARDS-Mut (MPtriVb), while the negative control group received saline (Saline). Each vaccine was formulated with either Al(OH)_3_ alone as a single adjuvant (S) or Al(OH)_3_ plus CpG as a dual adjuvant (D), resulting in the following groups: Saline-S, Saline-D, MPtriVa-S, MPtriVa-D, MPtriVb-S, and MPtriVb-D. The total protein content of each three-component vaccine was 15 μg per dose, with P1, P40/90, and CARDS-WT or CARDS-Mut mixed in a 1:1:1 mass ratio. The single adjuvant groups received 100 μg of Al(OH)_3_ per dose, while the dual adjuvant groups received 100 μg of Al(OH)_3_ plus 50 μg of CpG per dose. The immunization schedule was identical to that used for the single antigen immunogenicity assessment. Sera were collected 14 days after the final immunization and stored for subsequent analysis. The antigen dose (5 μg per mouse) and adjuvant doses were selected based on commonly used conditions reported in previous studies. The antigen dose (5 μg per mouse) and adjuvant doses were selected based on commonly used conditions reported in previous studies [19].

Specific IgG antibody titers in mouse sera were measured by indirect ELISA. Microtiter plates (Corning, NY, USA) were coated with 100 μL/well of antigen protein (P1, P40/90, CARDS-WT, or CARDS-Mut) diluted to 2 μg/mL in 50 mmol/L carbonate buffer (pH 9.6) and incubated overnight at 4 °C. Plates were washed twice with PBST (PBS containing 0.1% Tween-20), then blocked with 300 μL/well of 5% skim milk for 1 h at 37 °C. After blocking, 100 μL/well of serially diluted mouse serum was added and incubated for 1 h at 37 °C, followed by three washes with PBST. Next, 100 μL/well of HRP-conjugated goat anti-mouse IgG antibody (1:4000 dilution, Bioss Antibodies Co., Ltd., Beijing, China) was added, incubated for 1 h at 37 °C, and washed four times with PBST. TMB single-component substrate (100 μL/well, Solarbio Science & Technology Co., Ltd., Beijing, China) was added and incubated for 4 min at room temperature in the dark. The reaction was stopped by adding 50 μL/well of 2 mol/L H_2_SO_4_, and absorbance was measured immediately at 450 nm using a microplate reader. Antibody titers were defined as the highest serum dilution yielding an OD value ≥ 2.1 times that of the negative control.

### 2.6. Mycoplasma pneumoniae Challenge and Protective Efficacy Evaluation

Forty-eight BALB/c mice were grouped and immunized as described in Section 2.5 (three-component vaccine schedule). Fourteen days after the final immunization (day 42), mice were challenged with the *Mycoplasma pneumoniae* (MP) standard strain ATCC M129. Briefly, MP stock was revived and cultured in PPLO medium at 37 °C until the logarithmic growth phase. The bacteria were harvested by centrifugation, resuspended in sterile PBS, and administered intranasally to mice at a dose of 1 × 10^7^ CFU in 50 μL. Fourteen days post-challenge, mice were euthanized for sample collection and analysis: (1) Lung histopathology: Lungs from 3 mice per group were fixed in 4% paraformaldehyde for 24 h, embedded in paraffin, sectioned to 4 μm thickness, and stained with hematoxylin and eosin (H&E). Lung tissue sections were examined under a light microscope, and scoring was performed based on four dimensions: inflammatory cell infiltration in alveolar spaces or vascular walls, alveolar congestion, pulmonary hemorrhage, and alveolar wall thickening. The severity of each dimension was assessed in each sample, and scores were assigned accordingly. (2) Lung bacterial load: Left lung tissues (10 mg) from 5 mice per group were homogenized in 200 μL PBS. Total DNA was extracted, and MP-specific gene copy numbers were quantified by digital PCR. Results were expressed as CFU/μL. (3) Serum inflammatory cytokines: Blood was collected from the orbital sinus 14 days post-challenge. Sera were isolated, and concentrations of IL-6, TNF-α, and IFN-γ were measured using commercial ELISA kits.

### 2.7. Vaccine Safety Assessment

To systematically evaluate the safety of the MPtriV, BALB/c mice were randomly divided into 6 groups (Saline-S, Saline-D, MPtriVa-S, MPtriVa-D, MPtriVb-S, MPtriVb-D; n = 5 per group). Mice received a single immunization following the established protocol. Body weight and rectal temperature were monitored daily from day 0 to day 7 post-immunization. The body weight change rate was calculated relative to the day 0 weight. On day 7 post-immunization, serum was collected to assess liver and kidney function by measuring alanine aminotransferase (ALT), aspartate aminotransferase (AST), alkaline phosphatase (ALP), and blood urea nitrogen (BUN) levels using an automatic biochemical analyzer. Additionally, serum was collected on days 0, 1, 3, and 7 post-immunization to dynamically monitor systemic inflammation by measuring IL-1β, IL-6, TNF-α, and IFN-γ levels using a chemiluminescence detection system.

### 2.8. Statistical Analysis

All data were processed and visualized using GraphPad Prism version 10.3.0 (Windows 10). Each group contained n = 5/8 biological replicates. For multiple group comparisons, one-way ANOVA was applied, and for two-factor experiments, two-way ANOVA followed by Tukey’s multiple comparisons test was used. Although formal normality and homogeneity of variance tests were not performed, the distributions of the data were approximately normal and the variances were similar, making ANOVA appropriate. Statistical significance was defined as follows: *p* > 0.05 (not significant); *, *p* < 0.05; **, *p* < 0.01; ***, *p* < 0.001; and ****, *p* < 0.0001. All results are presented as mean ± standard deviation (Mean ± SD), and the error bars in the figures represent SD.

## 3. Results

### 3.1. Purification of P1, P40/90, CARDS-WT, and Four CARDS Mutant Recombinant Proteins

After prokaryotic expression and two-step purification had been performed, seven recombinant proteins (P1, P40/90, CARDS-WT, and four CARDS mutants) were successfully obtained. The theoretical molecular weights were 165 kDa for P1, 117 kDa for the P40/90 complex, and 69 kDa for CARDS-WT and its mutants. SDS-PAGE and Western blot analyses confirmed that the proteins migrated at their expected sizes (Figure 1B–H). Superdex™ 200 Increase 10/300 GL column (Cytiva, Uppsala, Sweden). analysis revealed single peaks for each protein, confirming their suitability for subsequent immunization experiments.

### 3.2. Recombinant Protein Was Determined to Be of High Purity

**Figure 2:**
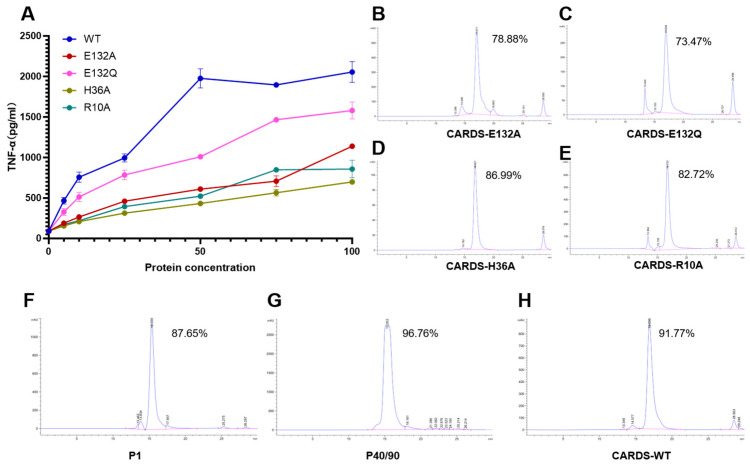
Toxicity screening of CARDS mutants and purity analysis of recombinant proteins. (**A**) Dose–response curves of TNF-α secretion from RAW264.7 cells treated with CARDS-WT and various mutants, used for screening attenuated mutants. (**B**–**H**) HPLC chromatograms of recombinant proteins on TSKgel G2000SWXL column. Purity values are indicated above the main peaks: (**B**) CARDS-E132A (78.88%), (**C**) CARDS-E132Q (73.47%), (**D**) CARDS-H36A (86.99%), (**E**) CARDS-R10A (82.72%), (**F**) P1 (87.65%), (**G**) P40/90 (96.76%), (**H**) CARDS-WT (91.77%).

HPLC analysis confirmed the high purity of all recombinant proteins (Figure 2B–H). The P1 protein showed a single main peak with a purity of 87.65% (Figure 2F), while the P40/90 complex exhibited an even higher purity of 96.76% (Figure 2G). For the CARDS toxin variants, wild-type CARDS (CARDS-WT) had a purity of 91.77% (Figure 2H). The four point mutants (E132A, E132Q, H36A, R10A) displayed purities ranging from 73.47% to 86.99% (Figure 2B–E), with the E132A mutant (CARDS-Mut) showing a purity of 78.88%. These results indicate that all recombinant proteins were successfully purified to levels adequate for subsequent immunological assays.

### 3.3. The Community-Acquired Respiratory Distress Syndrome (CARDS) Mutant Protein Was Obtained

To compare the cytotoxic differences between the wild-type (WT) CARDS protein and its mutants at the cellular level, RAW264.7 murine macrophages were stimulated with different recombinant proteins, and the levels of the pro-inflammatory cytokine TNF-α in the culture supernatants were quantified by ELISA. This approach enabled the evaluation of the inflammatory response induced by each protein. TNF-α is a key mediator of inflammation, and its secretion level is widely used as an indicator of cytotoxin-induced inflammatory activity.

The results are presented in Figure 2A. Upon stimulation of RAW264.7 cells with increasing concentrations of CARDS proteins, TNF-α levels in the supernatants were measured to assess the inflammatory potency of each protein. CARDS-WT induced TNF-α production at 5, 10, 25, 50, 75, and 100 μg/mL with levels of 465.9 ± 41.4 pg/mL, 756.2 ± 62.18 pg/mL, 994.4 ± 49.16 pg/mL, 1977 ± 117.7 pg/mL, 1895 ± 15.61 pg/mL, and 2054 ± 128.3 pg/mL, respectively, demonstrating a clear dose-dependent increasing trend. These findings indicate that CARDS-WT robustly stimulates macrophages to produce inflammatory responses, confirming its pronounced biological activity. At comparable TNF-α levels, differences in the required stimulation concentrations among proteins were observed. CARDS-E132Q induced a TNF-α level of 1009 ± 11.77 pg/mL at 50 μg/mL. In contrast, CARDS-WT required only 25 μg/mL to achieve a similar level (994.4 ± 49.16 pg/mL), indicating an approximately twofold reduction in inflammatory potency for E132Q. CARDS-E132A induced a TNF-α level of 609.6 ± 3.86 pg/mL at 50 μg/mL. By comparison, CARDS-WT achieved 465.9 ± 41.4 pg/mL at 5 μg/mL and 756.2 ± 62.18 pg/mL at 10 μg/mL, suggesting that the E132A mutant exhibits an approximately 5–10-fold reduction in cytotoxicity. CARDS-R10A and CARDS-H36A induced TNF-α levels of 521.8 ± 3.87 pg/mL and 432.7 ± 18.97 pg/mL, respectively, at 50 μg/mL, indicating an approximately 10-fold reduction in toxicity.

Collectively, these results demonstrate that the CARDS protein possesses strong inflammatory-inducing activity and that site-directed mutations at key amino acid residues can effectively attenuate its cytotoxicity. Notably, CARDS toxin exerts its catalytic function through the highly conserved “R–STS–E” motif within its N-terminal ADP-ribosyltransferase (ADPRT) domain. Glutamate at position 132 (E132), as a core residue of this motif, directly participates in NAD^+^ binding and catalysis and serves as a critical “switch” residue for initiating ADP-ribosylation. The functional importance of E132 has been well established at the molecular level. In this study, mutations at this position (E132A and E132Q) markedly reduced the inflammatory activity of the toxin, further underscoring the pivotal role of E132 in CARDS toxin function. Therefore, subsequent investigations will focus on E132A (with pronounced attenuation) for in-depth analysis.

### 3.4. Three-Component Vaccine (MPtriV) Exhibits Robust Immunogenicity

**Figure 3:**
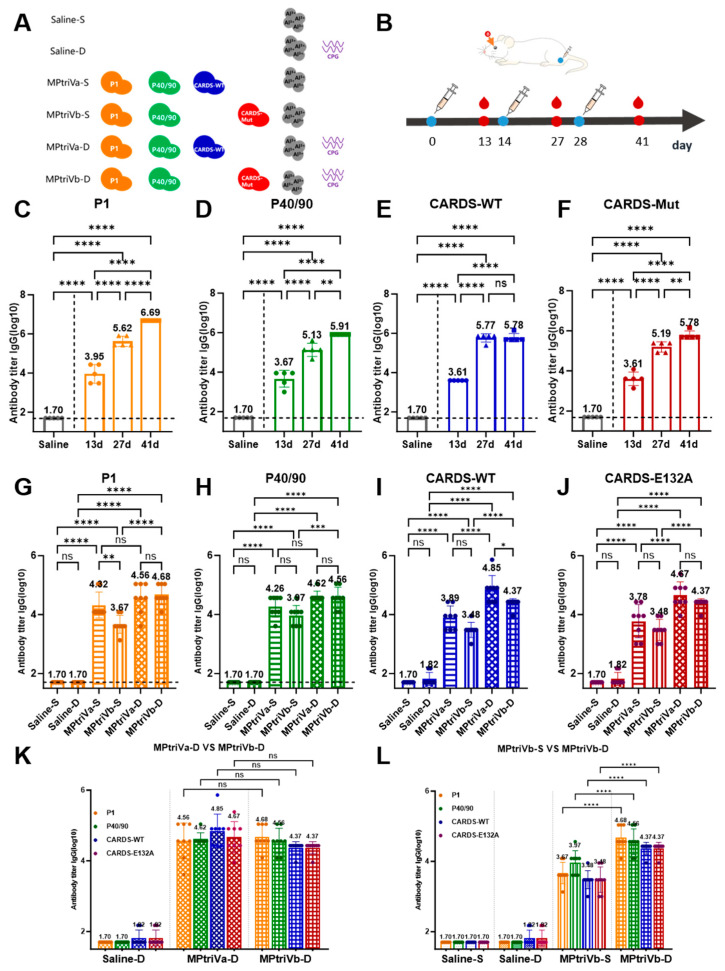
Formulation, immunization schedule, and antigen-specific IgG antibody responses of the three-component *M. pneumoniae* vaccine. (**A**) Formulation scheme showing the 6 experimental groups, their respective vaccine and adjuvant combinations. (**B**) Immunization schedule showing time points for immunizations (days 0, 14, 28) and serum collection (days 13, 27, 41). (**C**–**F**) Serum titers (log10) of specific IgG antibodies against P1, P40/90, CARDS-WT, and CARDS-Mut following single antigen immunizations (days 0, 14, 28) and serum collection (days 13, 27, 41). The saline group served as the negative control. (**G**–**J**) Serum antibody titers (days 41) against P1, P40/90, CARDS-WT, and CARDS-Mut in mice immunized according to the groups shown in (**A**). (**K**) Comparison of antibody titers (days 41) between the MPtriVa-D group (containing CARDS-WT with dual adjuvant) and the MPtriVb-D group (containing CARDS-Mut with dual adjuvant). (**L**) Comparison of antibody titers (days 41) between the MPtriVb-S (single adjuvant) and MPtriVb-D (dual adjuvant) groups. Data are presented as mean ± SD. *p* > 0.05 (not significant); *, *p* < 0.05; **, *p* < 0.01; ***, *p* < 0.001; ****, *p* < 0.0001.

To evaluate the baseline immunogenicity of each antigen component, mice were immunized individually with P1, the P40/90 complex, CARDS-WT, or CARDS-Mut, each formulated with Al(OH)_3_ adjuvant. Serum samples were collected after three immunizations, and specific IgG antibody titers were measured. As shown in Figure 3C–F, the P1 protein induced the highest antibody titers, reaching 4.9 × 10^6^. The P40/90 complex induced titers of 8.1 × 10^5^. Both CARDS-WT and CARDS-Mut induced comparable antibody titers of 6.0 × 10^5^, with no significant difference observed between them. All antigen-immunized groups exhibited significantly higher antibody titers compared to the saline control group (titer approximately 5 × 10^1^), demonstrating that P1, P40/90, CARDS-WT, and CARDS-Mut each possess strong immunogenicity. Importantly, CARDS-Mut retained full immunogenicity despite the significant reduction in toxicity achieved by the E132A point mutation, successfully achieving the initial goal of “attenuation without compromising immunogenicity.”

While these results demonstrate that each individual antigen can effectively activate humoral immunity, a single-antigen vaccine can only target one aspect of *Mycoplasma pneumoniae* (MP) pathogenesis (e.g., P1 blocking adhesion or CARDS neutralizing the toxin), making it difficult to achieve broad-spectrum protection. To achieve more comprehensive immune protection, we formulated three-component vaccines by combining P1 and P40/90 with either CARDS-WT (MPtriVa) or CARDS-Mut (MPtriVb). Mice were immunized with these vaccines, adjuvanted with either Al(OH)_3_ alone or Al(OH)_3_ plus CpG, and sera were analyzed for specific IgG antibodies against P1, P40/90, and the respective CARDS antigen. As shown in Figure 3G–J, immunization with MPtriVa or MPtriVb simultaneously elicited high levels of antibodies against P1, P40/90, and the respective CARDS antigen, demonstrating that the vaccines successfully induced a broad-spectrum humoral immune response targeting multiple antigens. Serum antibody levels against MPtriVa and MPtriVb were measured at Day 41 post-immunization. In contrast to single-antigen immunization, which only induces antibodies against a single target and fails to address other pathogenic factors, this approach expanded the immune protection spectrum while preserving the immunogenicity of each individual component.

To verify whether the attenuated CARDS-Mut retains its ability to effectively induce an immune response within the vaccine formulation, we compared the antibody levels induced by the three-component vaccines containing CARDS-WT (MPtriVa) and CARDS-Mut (MPtriVb). As shown in Figure 3K, under identical adjuvant conditions, the MPtriVa and MPtriVb groups exhibited comparable antibody titers against P1 (3.6 × 10^4^ and 4.8 × 10^4^, respectively) and against P40/90 (4.2 × 10^4^ and 3.6 × 10^4^, respectively), with no significant differences observed between the two groups. With respect to CARDS-specific antibodies, the MPtriVb-D group exhibited identical antibody titers of 2.3 × 10^4^ against both CARDS-WT and CARDS-Mut. In the MPtriVa-D group, the antibody titers against CARDS-WT and CARDS-Mut were 7.1 × 10^4^ and 4.7 × 10^4^, respectively, with no statistically significant differences observed between groups. These results indicate that CARDS-Mut retains strong immunogenicity within the vaccine formulation and that the E132A mutation does not impair immune recognition of antigenic epitopes shared between CARDS-WT and CARDS-Mut.

Furthermore, this study systematically evaluated the impact of different adjuvant combinations on immunogenicity. As shown in Figure 3L, the Al(OH)_3_ + CpG dual-adjuvant formulation significantly enhanced antibody responses across all immunization groups. Taking the CARDS-E132A–containing vaccine (MPtriVb) as an example, the dual-adjuvant group exhibited an anti-P1 antibody titer of 4.8 × 10^4^, compared with 3.7 × 10^3^ in the single-adjuvant group, representing an approximately 10-fold increase (*p* < 0.0001). For P40/90, antibody titers increased from 9.3 × 10^3^ to 4.9 × 10^4^, corresponding to an approximately 5-fold enhancement (*p* < 0.0001). Similarly, antibody titers against both CARDS-WT and CARDS-E132A increased from 3.0 × 10^3^ to 2.3 × 10^4^, representing an approximately 10-fold increase. A comparable trend was observed in the MPtriVa group. These findings indicate that the combination of Al(OH)_3_ and CpG exerts a synergistic adjuvant effect, markedly enhancing the magnitude of humoral immune responses elicited by the vaccine.

In summary, the single-antigen immunogenicity data confirmed that P1, P40/90, CARDS-WT, and CARDS-Mut, when formulated with Al(OH)_3_ adjuvant, all induce high levels of specific IgG antibodies in mice. Notably, CARDS-Mut retained full immunogenicity while exhibiting significantly reduced toxicity, providing a solid foundation for multi-component vaccine development. Building on this, the three-component vaccine MPtriV successfully induced broad-spectrum humoral immunity against P1, P40/90, and CARDS, demonstrating that the multi-antigen combination strategy expands the immune protection profile while preserving the immunogenicity of each component. Importantly, the antibody levels induced by the CARDS-Mut-containing vaccine (MPtriVb) were comparable to those induced by the CARDS-WT-containing vaccine (MPtriVa), further validating the success of the E132A site-directed mutagenesis strategy for “attenuation without compromising immunogenicity.” Adjuvant effect analysis showed that the Al(OH)_3_ + CpG dual adjuvant significantly enhanced vaccine immunogenicity, increasing antigen-specific antibody titers by approximately 6–10 fold compared to the single adjuvant.

### 3.5. Three-Component Vaccine (MPtriV) Significantly Attenuates Mycoplasma pneumoniae Challenge-Induced Lung Injury, Reduces Pulmonary Bacterial Load, and Suppresses Inflammatory Cytokine Secretion

**Figure 4:**
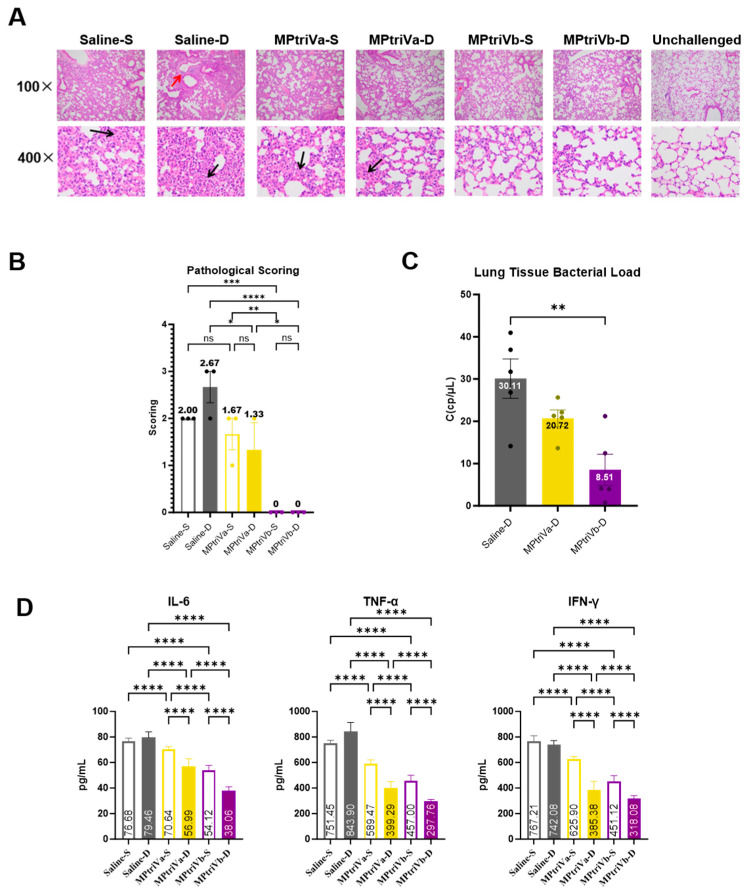
Lung histopathology, pathological scores, bacterial loads, and inflammatory cytokine levels in vaccinated mice post-MP challenge. (**A**) Representative H&E-stained lung sections. Black arrows indicate mild alveolar wall thickening; red arrows indicate peribronchial inflammatory cell infiltration. (**B**) Lung histopathology scores (0–4 scale). (**C**) Pulmonary bacterial loads measured by digital PCR (CFU/μL). (**D**) Serum levels of inflammatory cytokines (IL-6, TNF-α, IFN-γ) were measured by ELISA. Data are presented as mean ± SD. Statistical analysis was performed using one-way ANOVA. *p* > 0.05 (not significant); *, *p* < 0.05; **, *p* < 0.01; ***, *p* < 0.001; ****, *p* < 0.0001.

After challenge, lung tissues from each group of mice were subjected to H&E staining and pathological scoring to evaluate the lung protection effect of the vaccine. The results showed that MPtriVb exhibited the best protection, with a pathological score of 0, indicating that the lung tissue structure was nearly normal under the microscope, with no apparent inflammatory cell infiltration or thickening of the alveolar walls (Figure 4A). This suggests that this vaccine combination can completely alleviate the lung tissue damage caused by MP infection in mice. In contrast, the MPtriVa group containing wild-type CARDS-WT showed relatively weaker protection. Under single-adjuvant conditions, the MPtriVa-S group had a pathological score of 1.67, with a small amount of inflammation in the alveolar walls (Figure 4B). Under dual-adjuvant conditions, the MPtriVa-D group had a pathological score of 1.33, with slightly reduced inflammation compared to the MPtriVa-S group, but the protection was still not complete. This indicates that although the MPtriVa vaccine can partially alleviate lung damage, its protective effect is limited and cannot fully resolve the injury. As a negative control, the saline-adjuvanted groups (Saline-S and Saline-D) exhibited typical inflammatory damage post-MP infection: the Saline-S group showed significant thickening of the alveolar walls under the microscope (Figure 4A, black arrow) and a pathological score of 2.00; the Saline-D group showed extensive infiltration of inflammatory cells around the bronchi (Figure 4A, red arrow) and a pathological score of 2.67. No significant difference in damage was observed between the two groups, suggesting that the adjuvants alone do not alleviate MP-induced lung inflammation. Further analysis of the adjuvant type’s effect on protection showed that its effect varied depending on the type of CARDS protein in the vaccine. For the CARDS-WT-containing vaccine, dual-adjuvant (MPtriVa-D) showed a slight reduction in the pathological score compared to the single-adjuvant group (1.33 vs. 1.67), but it did not provide complete protection. However, for the CARDS-Mut-containing vaccine, which provided complete protection, the single-adjuvant (MPtriVb-S) was sufficient to fully alleviate lung tissue damage, and the dual-adjuvant (MPtriVb-D) maintained the same 0 score without further enhancing the protection. These results indicate that the excellent “attenuation without compromising immunogenicity” feature of CARDS-Mut is key to achieving complete lung protection. In conclusion, MPtriVb can completely alleviate the lung tissue pathological damage caused by MP infection in mice.

To assess the vaccine’s ability to inhibit *Mycoplasma pneumoniae* (MP) lung colonization, the lung bacterial load was measured 14 days post-challenge in mice from each group. Based on the previous pathological results, this section focuses on comparing the bacterial load differences between the dual-adjuvant groups. The results showed that the MPtriVb-D group exhibited the most prominent inhibition of MP lung colonization, with a bacterial load of only 8.51 CFU/μL (Figure 4C), which was significantly lower than that of the saline control group (*p* < 0.01). The MPtriVa-D group had a bacterial load of 20.72 CFU/μL, approximately 2.4 times higher than that of MPtriVb-D, indicating that this vaccine could partially inhibit MP proliferation but was less effective than MPtriVb-D. As a negative control, the saline-D group had the highest bacterial load, reaching 30.11 CFU/μL (Figure 4C), suggesting substantial MP colonization and proliferation in the lungs of non-immunized mice, which is a key factor in the inflammatory damage following infection. The bacterial load results were highly consistent with the previous pathological damage assessment: the MPtriVb-D group had the lowest bacterial load (8.51 CFU/μL) and the pathological score of 0 (complete protection); the MPtriVa-D group had a moderate bacterial load (20.72 CFU/μL) and a pathological score of 1.33 (partial protection); and the saline-D group had the highest bacterial load (30.11 CFU/μL) with a pathological score of 2.67 (severe damage). A trend was observed in which reductions in bacterial load were generally accompanied by alleviation of lung tissue inflammation, further confirming the significant advantage of MPtriVb-D in reducing pathogen burden and alleviating infection-induced damage.

The secretion levels of three core inflammatory cytokines (IL-6, TNF-α, IFN-γ) were measured to evaluate the vaccine’s ability to alleviate lung inflammation following MP infection. The results showed that the MPtriVb-D group exhibited the most significant inhibition of inflammatory cytokine secretion, with the lowest levels of IL-6, TNF-α, and IFN-γ among all vaccine groups (Figure 4D), which was highly consistent with the pathological scoring and bacterial load results. Further comparison of the effects of different vaccine combinations revealed that MPtriVb-D and MPtriVb-S were significantly more effective in suppressing these cytokines than MPtriVa-D and MPtriVa-S (Figure 4D), indicating that CARDS-Mut, due to its reduced toxicity, performed better in alleviating inflammation. The impact of adjuvant type was also significant: regardless of the CARDS protein included, the dual-adjuvant groups had lower cytokine levels than their corresponding single-adjuvant groups, suggesting that the Al(OH)_3_ + CpG dual adjuvant further enhanced the vaccine’s anti-inflammatory effect. In the saline-adjuvanted control group, the levels of all three cytokines were significantly elevated (Figure 4D), reflecting the intense inflammatory response in the lungs following MP infection. In contrast, the inflammatory cytokine levels in all other immunized groups were significantly lower than those in the saline control group (*p* < 0.0001), confirming that the vaccine effectively suppressed the inflammation induced by MP infection. In conclusion, the MPtriVb-D group showed the best results in suppressing cytokine secretion, and this finding, in conjunction with the lung tissue pathology and bacterial load assessments, forms a comprehensive evidence chain that demonstrates the significant protective advantage of this vaccine combination against MP infection.

### 3.6. Three-Component Vaccine (MPtriV) Exhibits a Favorable Safety Profile

To systematically evaluate the safety of MPtriV, the general physiological status, vital organ function, and systemic inflammatory responses of mice were dynamically monitored following immunization.

**Figure 5:**
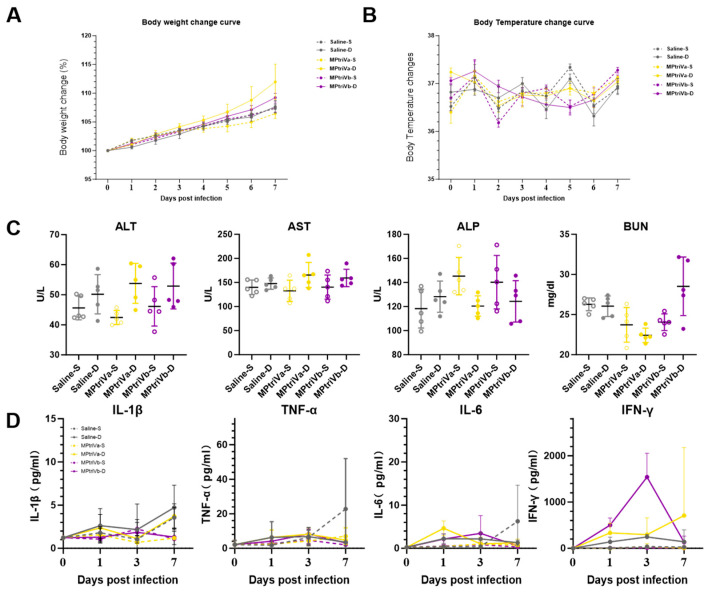
Dynamic changes in body weight, body temperature, liver/kidney function, and inflammatory cytokines post-immunization for vaccine safety assessment. (**A**) Body weight change curve from day 0 to day 7 post-immunization. (**B**) Body temperature change curve over the same period. (**C**) Serum levels of ALT, AST, ALP, and BUN on day 7 post-immunization, reflecting liver and kidney function. (**D**) Serum levels of IL-1β, IL-6, TNF-α, and IFN-γ on days 0, 1, 3, and 7 post-immunization, dynamically monitoring systemic inflammation.

Regarding general physiological status, all groups exhibited steady weight gain from day 0 to day 7 post-immunization (Figure 5A). Body temperature remained within the normal physiological range throughout the monitoring period (Figure 5B). No statistically significant differences in body weight change or body temperature fluctuations were observed between any of the vaccine groups (MPtriVa-S, MPtriVa-D, MPtriVb-S, and MPtriVb-D) and the saline control groups (Saline-S, Saline-D). These results indicate that the vaccine immunization did not adversely affect the baseline physiological condition of the mice.

Regarding vital organ function, analysis of serum liver and kidney function markers on day 7 post-immunization revealed that levels of alanine aminotransferase (ALT), aspartate aminotransferase (AST), alkaline phosphatase (ALP), and blood urea nitrogen (BUN) were within normal reference ranges for all groups (Figure 5C). No statistically significant differences were observed between any vaccine group and the saline control groups, suggesting that MPtriV immunization did not cause significant liver or kidney damage.

Regarding systemic inflammation, dynamic monitoring of serum IL-1β, IL-6, TNF-α, and IFN-γ levels on days 0, 1, 3, and 7 post-immunization showed slight fluctuations in all groups on days 1–3, with levels gradually returning to baseline thereafter (Figure 5D). At all time points, no statistically significant differences were observed between any vaccine group and the saline control groups. This indicates that the inflammatory response induced by the vaccine was comparable to that induced by saline alone, and the vaccine did not trigger excessive systemic inflammation.

In summary, following MPtriVb immunization, no significant differences were observed in mouse body weight, body temperature, liver and kidney function markers, or inflammatory cytokine levels compared to the saline control groups. All safety parameters remained within normal ranges, indicating that the MPtriVb vaccine has a favorable safety profile and providing solid evidence for further research.

## 4. Discussion

*Mycoplasma pneumoniae* infection remains a significant global public health challenge, placing a substantial disease burden, particularly on children and adolescents [20,21]. This study utilized the well-defined components, high safety, and strong specificity of subunit vaccines, selecting key MP virulence factors—attenuated CARDS toxin and the P1 and P40/90 antigens—as targets to construct a three-component recombinant subunit vaccine, designated MPtriV.

CARDS toxin, the sole virulence factor of *Mycoplasma pneumoniae* (MP), plays a central role in its pathogenesis. It exerts its catalytic function via a highly conserved “R–STS–E” motif within its N-terminal ADP-ribosyltransferase (ADPRT) domain. Glutamate at position 132 (E132), a critical residue within this motif, directly participates in NAD^+^ binding and catalysis, acting as a key “switch residue” to initiate the ADP-ribosyltransferase reaction, as substantiated by multiple studies [12]. However, this potent enzymatic activity also underpins CARDS toxin’s ability to induce robust inflammatory responses, with its high toxicity being the primary obstacle to incorporating it into vaccine designs. Therefore, attenuating its toxicity while preserving its immunogenicity is essential for realizing its potential as a vaccine antigen.

To address this, we designed four site-directed CARDS toxin mutants targeting key functional residues and screened their toxicity by measuring TNF-α secretion in RAW264.7 cells. All mutants exhibited significantly reduced toxicity compared to the wild-type. Notably, the H36A, R10A, and E132A mutants demonstrated the greatest attenuation, inducing TNF-α levels approximately one-tenth of the wild-type levels. Considering both the molecular mechanistic evidence and toxicity data, we ultimately selected the E132A mutant (CARDS-Mut) for further experiments. This decision was based on two key factors: first, E132 is the core residue in the “R–STS–E” motif, directly initiating catalysis, with its functional importance well-documented at the molecular level; second, the E132A mutant exhibited attenuation comparable to H36A and R10A in the toxicity screen. Subsequent single-antigen immunization experiments confirmed that CARDS-Mut induced antibody titers equivalent to the wild-type, demonstrating that the E132A mutation effectively abrogates the toxin’s enzymatic activity without compromising key antigenic epitopes. This successfully achieved “attenuation without compromising immunogenicity.” These results provide direct experimental evidence to address safety concerns regarding the use of CARDS toxin as a vaccine antigen, in line with the classical vaccine development strategy of modifying virulence factors to improve safety.

Subunit vaccines often exhibit weak immunogenicity, making the selection of an appropriate adjuvant critical for enhancing immune responses [22]. In this study, mice were immunized with either MPtriVa or MPtriVb vaccines formulated with Al(OH)_3_ alone or with the Al(OH)_3_ + CpG dual adjuvant. All immunized groups, regardless of vaccine composition, showed significantly higher antibody titers compared to saline controls, confirming that the three-component vaccines effectively induce specific immune responses. Furthermore, regardless of whether the vaccine contained CARDS-WT or CARDS-Mut, the dual adjuvant groups exhibited markedly superior immunogenicity compared to the single adjuvant groups. Al(OH)_3_ enhances humoral immunity by adsorbing antigens and prolonging their retention at the injection site, while CpG activates Toll-like receptor 9 (TLR9) to induce a Th1-biased cellular immune response [23]. This combination synergistically enhances both humoral and cellular immunity, aligning with previous studies that demonstrated superior immune enhancement with the Al(OH)_3_ + CpG combination compared to single adjuvants [24]. This finding supports Al(OH)_3_ + CpG as an ideal adjuvant combination for MP subunit vaccines.

Compared to previously reported *Mycoplasma pneumoniae* (MP) vaccines, the three-component dual-adjuvant MPtri vaccine constructed in this study offers significant comprehensive advantages. Early inactivated MP vaccines, while simple to prepare, were characterized by weak immunogenicity, requiring multiple doses, and provided protection lasting only 6–12 months [13]. Live-attenuated vaccines, although capable of inducing comprehensive immune responses, carry inherent safety risks due to the potential for reversion to virulence [20]. In contrast, our subunit vaccine avoids the redundant pathogenic factors present in whole-cell vaccines by precisely selecting key antigens and modifying the CARDS toxin. The E132A mutation effectively eliminates the CARDS toxin’s high toxicity, and post-immunization, mice showed no abnormalities in liver or kidney function and only minimal systemic inflammation, indicating a safety profile superior to that of whole-cell vaccines. The dual adjuvant formulation induced specific IgG titers reaching 4.8 × 10^4^, and post-challenge, pulmonary bacterial load was reduced by 71.7%. Existing protein subunit vaccines, such as HP14/30 or P1C DNA vaccines, typically focus solely on adhesion proteins, which often result in persistent pulmonary inflammation and difficulty in inducing effective mucosal immunity post-challenge [18,25]. In contrast, our three-antigen combination targets both the adhesion and toxin-mediated damage pathways. CARDS-Mut synergizes with P1 and P40/90 to achieve “adhesion blockade + inflammation suppression,” resulting in a post-challenge pathology score of 0 and demonstrating superior protective efficacy. Previous vaccines containing wild-type CARDS toxin were hindered by safety concerns due to excessive inflammatory responses [11,15]. Our study addresses this issue by demonstrating that site-directed mutagenesis reduces CARDS toxin toxicity by 90% while retaining its immunogenicity. Regarding adjuvants, while single aluminum adjuvants primarily enhance humoral immunity, single CpG adjuvants have limited antigen retention capacity [9,16]. The Al(OH)_3_ + CpG dual adjuvant formulation in this study synergistically amplifies the immune response while concurrently suppressing excessive inflammatory cytokine secretion, leading to superior immune enhancement compared to existing single-adjuvant formulations. Furthermore, our prokaryotically expressed subunit vaccine relies on mature, cost-effective production processes, making it more suitable for large-scale population immunization. This offers a more viable path toward clinical translation for MP vaccines compared to nucleic acid or live vector vaccines, which are still in early research stages and have limited clinical translation potential [9,18].

The results of this study support our initial hypotheses. The three-component vaccine demonstrated enhanced protective efficacy, highlighting the advantage of the multi-antigen strategy. The E132A mutant of CARDS toxin achieved effective attenuation while retaining immunogenicity, confirming the feasibility of this design. Furthermore, the combination of Al(OH)_3_ and CpG adjuvants enhanced the immune response, supporting their synergistic effect.

This study has several limitations. First, we only evaluated two adjuvants: the Al(OH)_3_ single adjuvant and the Al(OH)_3_ + CpG dual adjuvant. It remains unclear whether other adjuvant types, such as nano-adjuvants or mucosal adjuvants, could further optimize the immune response, particularly by enhancing mucosal immunity and promoting long-lasting immune memory. A systematic comparison and screening of various adjuvants is needed to address this gap. Second, while the introduction of the attenuated CARDS mutant is a central aspect of this study, the long-term safety profile of CARDS-Mut within the vaccine still requires in-depth evaluation. Although our preliminary data demonstrate significantly reduced toxicity and retained immunogenicity for the E132A mutant, it is essential to assess whether the attenuated CARDS protein might induce unexpected immunopathological reactions in vivo. Additionally, the safety profile following long-term immunization and whether the immunogenicity or safety characteristics change when combined with different adjuvants remains under-investigated. These aspects warrant further investigation using extended observation periods and diverse animal models.

## 5. Conclusions

In conclusion, this study successfully developed a three-component recombinant subunit vaccine, MPtriV, targeting *Mycoplasma pneumoniae* (MP). The vaccine incorporates a CARDS toxin mutant (CARDS-Mut) with markedly reduced toxicity, generated through site-directed mutagenesis. When formulated with the dual adjuvant Al(OH)_3_ + CpG, MPtriV demonstrated optimal immunogenicity and protective efficacy. This strategy effectively addresses the safety concerns associated with using CARDS toxin as a vaccine antigen while eliciting robust immune responses against MP infection. Overall, our findings provide a novel strategy and experimental foundation for the development of safe and effective MP subunit vaccines, offering strong support for future translational research.

## Data Availability

The raw data supporting the conclusions of this article will be made available by the authors, without undue reservation.
